# The impact of ADL disability in middle-aged and older adults on the incidence of hip fractures and the mediating role of depression: a longitudinal evidence from CHARLS

**DOI:** 10.3389/fmed.2025.1604729

**Published:** 2025-07-11

**Authors:** Chenyang Li, Congcong Luo, Jiayi Zhu, Ruonan You, Qiang Yuan, Ning Zhang, Ying Zhang

**Affiliations:** 1 Department of College of Orthopedics and Traumatology, Fujian University of Traditional Chinese Medicine, Fuzhou, Fujian, China; 2 Luoyang Orthopedic-Traumatological Hospital of Henan Province (Henan Provincial Orthopedic Hospital), Luoyang, China; 3 Department of Graduate School of Henan University of Chinese Medicine, Henan University of Traditional Chinese Medicine, Zhengzhou, Henan, China; 4 Zhengzhou Health College, Zhengzhou, Henan, China

**Keywords:** hip fractures, Activities of Daily Living (ADL), depressive symptoms, mediating role, older adults

## Abstract

**Background:**

Hip fractures pose a major public health burden in aging populations, driven by rising osteoporosis prevalence and demographic aging. In China, nearly 2 million new cases occurred in 2019, disproportionately affecting middle-aged and older women. Despite extensive research on biomedical risk factors, mechanistic links between Activities of Daily Living (ADL) disability and hip fractures remain unclear.

**Objectives:**

This study aims to elucidate the relationship between ADL disability and hip fractures incidence among middle-aged and older adult individuals in China. Furthermore, it seeks to explore the potential mediating role of depressive symptoms in this association, thereby contributing to a deeper understanding of hip fracture risk determinants.

**Methods:**

This longitudinal analysis utilized data from the China Health and Retirement Longitudinal Study (CHARLS) covering the period from 2011 to 2015. The study involved 5,066 participants who had no pre-existing hip fractures at baseline. ADL functional capacity was assessed by categorizing Instrumental Activities of Daily Living (IADL) and Basic Activities of Daily Living (BADL). Depressive symptoms were measured using the Center for Epidemiological Studies Depression Scale (CES-D-10). The incidence of hip fractures was documented over a four-year follow-up period, and both logistic regression and mediation analyses were performed to investigate the associations.

**Results:**

Within the follow-up cohort, a total of 256 incident hip fractures were recorded. After adjusting for confounding variables, IADL disability emerged as a significant predictor of fracture risk (OR = 1.42, 95% CI: 1.07–1.89, *p* = 0.017), while BADL disability was found to have an even greater impact (OR = 1.96, 95% CI: 1.47–2.61, *p* < 0.001). Mediation analysis indicated that depressive symptoms accounted for a substantial portion of the association, mediating 43.8% of the relationship between IADL disability and hip fractures risk, and contributing to 23.4% of the association between BADL disability and hip fractures.

**Conclusion:**

ADL disability, particularly BADL, independently predicts hip fractures in older Chinese adults. Depressive symptoms mediate 43.8% of the risk associated with IADL and 23.4% of the effect related to BADL. Therefore, integrating depression management into ADL-focused interventions may enhance strategies for fracture prevention.

## 1 Introduction

Hip fractures are a serious public health issue, often leading to severe complications and death, particularly among the older adult. With the acceleration of population aging and the rising prevalence of osteoporosis, the incidence of hip fractures in the older adult population continues to increase (1–3). According to relevant data from 2019, there were approximately 2 million new cases of hip fractures in China, with an age-standardized incidence rate of 117.8 per 100,000 people. This trend is expected to continue with population aging, posing a greater burden on healthcare systems and society (4). The older adult, especially women, face a higher risk of hip fractures, particularly among individuals aged ≥80 years (5, 6).

Previous research has primarily concentrated on biomedical factors, such as osteoporosis. However, there is a notable lack of systematic studies examining the mechanisms that link disability in ADL with hip fractures. ADL encompasses Basic Activities of Daily Living (BADL), such as getting up and washing hands, as well as Instrumental Activities of Daily Living (IADL), such as grocery shopping and housework (7). Disability in ADL significantly impacts the quality of life for middle-aged and older adult individuals in China. ADL disability is associated with various negative outcomes, often resulting in a decline in individual quality of life (8). Research indicates that a majority of older adult individuals experience a decline in functional abilities related to daily activities, with the incidence rate of ADL disability among the older adult being approximately 17.22 per thousand. Given the increasing trend of an aging population in China, it is projected that by 2060, the number of individuals aged 65 and above with ADL disability will rise to 96.2 million (8, 9).

A meta-analysis indicates that older adults in Europe exhibit significantly lower scores in ADL prior to hip fractures compared to their non-fractured peers, suggesting that functional decline may precede fractures. The Health and Retirement Study (HRS) confirms that many patients with hip fractures had already experienced severe disability before the fracture, with this disability accelerating in the lead-up to the event. Another HRS study highlights that many older adults relied on others for daily activities prior to their fractures, facing mobility issues and difficulties with stair climbing (10–12). Depressive symptoms may further link ADL disability to fracture risk via psychobiological pathways. Depression is highly prevalent among the older adult, with its incidence closely linked to health status; poorer health conditions correlate with a higher incidence of depression (13, 14). Research demonstrates a bidirectional relationship between ADL disability and depressive symptoms, indicating that older adults with ADL disability are more likely to exhibit depressive symptoms, while depressive symptoms can also adversely affect ADL performance (15–17), depression has been established as an independent risk factor for hip fractures, with numerous studies finding that individuals with depressive symptoms are more susceptible to hip fractures (18–21).

We hypothesize that there exists a significant relationship between ADL disability and hip fractures in the older adult, with the potential for depressive states to mediate this relationship. This study employs longitudinal data from the China Health and Retirement Longitudinal Study (CHARLS) to investigate the correlation between ADL disability and the incidence of hip fractures, as well as to assess the degree to which depressive symptoms mediate this association among older adults in China.

## 2 Method

### 2.1 Study population

The CHARLS is a comprehensive longitudinal survey aimed at collecting extensive health-related data from mainland Chinese residents aged 45 and older. It encompasses a wide array of inquiries concerning economic status, physical and mental health, demographics, and the social networks of the aging population. The baseline survey, conducted in 2011, utilized a stratified multistage sampling method, covering 150 counties or urban districts across 28 provinces and involving 17,708 individuals from 10,257 households (8, 22). We conducted a longitudinal study over 4 years (2011–2015), using the 2011 population data as the baseline. After excluding subjects diagnosed with hip fractures at baseline or lacking information on hip fractures during follow-up, we monitored and observed eligible subjects until 2015.

**Figure 1:**
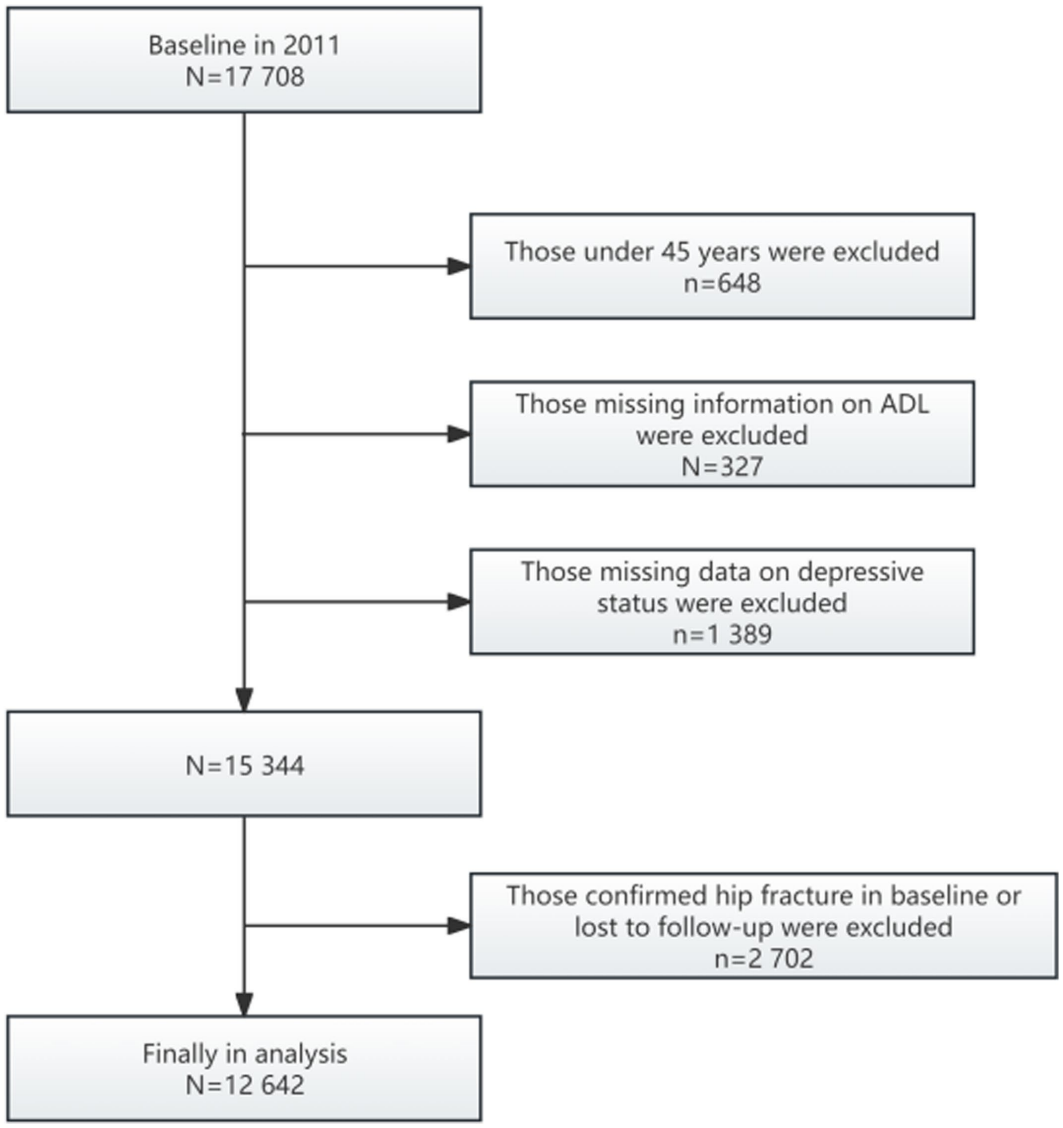
Flow chart of sample selection and the exclusion criteria. According to the figure, the number of observation was 12,642.

We conducted a four-year longitudinal study (2011–2015), using the population data from 2011 as the baseline. Among the initially screened 17,708 participants, 12,642 were excluded due to the following reasons: individuals under 45 years old (*n* = 648), missing ADL information (*n* = 327), absence of CES-D-10 score data (*n* = 1,389), incomplete hip fracture information in Wave 1 (*n* = 9) and Wave 3 (*n* = 2,485), and baseline diagnosed hip fractures (*n* = 208). Ultimately, the longitudinal analysis included 12,642 participants (Figure 1), comprising 12,386 individuals without hip fractures and 256 individuals with hip fractures.

The CHARLS survey was approved by the Biomedical Ethics Committee of Peking University (IRB00001052-11015) and obtained written informed consent from all participants before their involvement in this survey. This study adhered to the Strengthening the Reporting of Observational Studies in Epidemiology (STROBE) reporting guidelines (23).

### 2.2 Assessment of hip fractures

During the household interviews, each participant was asked the following question in the baseline survey: “Have you ever had a fracture?” To ensure a clear understanding of the definition of hip fractures, the interviewer provided a detailed explanation of the location of the hip bone. Participants who responded “Yes” to this question were classified as having experienced hip fractures. Incident hip fractures were defined as participants who did not have hip fractures at baseline but were identified as having one during the follow-up survey (24, 25).

### 2.3 Assessment of ADL disability

We assessed ADL disability using the BADL and IADL scales. BADL encompasses six items measuring dressing, bathing, eating, getting up, toileting, and continence. IADL consists of five items, including housework, cooking, shopping, taking medication, and managing finances. Each item offers four response options: (1). No difficulty; (2). Difficulty but achievable; (3). Some difficulty, requires assistance; and (4). Unable to complete. Based on prior studies, the outcome is dichotomous; the lack of complete independence in any ADL item is defined as having an ADL disability (26, 27).

### 2.4 Depressive symptoms

The Center for Epidemiological Studies Depression Scale (CES-D-10) consists of 10 items designed to assess the depressive symptoms experienced by informants over the previous week. This scale includes two parts: eight negative items and two positive items. Negative moods or behaviors occurring for less than 1 day, 1 to 2 days, 3 to 4 days, or 5 to 7 days are rated as 0, 1, 2, or 3, respectively. The two positive items are scored in reverse (28, 29). The scores from all 10 items are summed to obtain a total score, which ranges from 0 to 30; a higher score indicates more severe depressive symptoms. Individuals with a score of ≥10 are classified as having depressive symptoms, while those with a score of < 10 are considered normal (30).

### 2.5 Evaluation of covariates

For the selection of covariates, we referred to previous studies (31, 32) following baseline characteristics were extracted: age, sex, residence (rural or urban), education level (below primary, primary, secondary, high school and above), illiteracy, primary school, middle school, high school or above, marital status (married or others), sleep duration, drinking status (ex-drinking, current drinking, or never drinking), smoking status (ex-smoking, current smoking, or never smoking), and Body Mass Index (BMI). Chronic diseases assessed included hypertension and diabetes. BMI is calculated as weight (kg) divided by the square of height (m). Participants are classified as having hypertension if they self-report a physician-diagnosed hypertension or have a systolic blood pressure (BP) ≥ 140 mmHg or diastolic BP ≥ 90 mmHg. Diabetes is defined as self-reported physician-diagnosed diabetes, fasting plasma glucose level ≥ 7 mmol/L, random plasma glucose level ≥ 11.1 mmol/L, or glaciated hemoglobin (HbA1c) ≥ 6.5%. The assessment of fall history was based on the question, “Have you fallen in the past 2 years?” Participants who answered “Yes” to this question were considered to have fallen in the past 2 years (33).

### 2.6 Statistical analysis

In the dataset, we described the baseline characteristics of non-normally distributed data: categorical variables are presented as totals and percentages, while continuous variables are represented by medians and interquartile ranges (IQR). Inter-group comparisons were performed using *t*-tests, Mann–Whitney U tests, or chi-square tests, depending on the type of data distribution. We employed logistic regression analysis to examine the prospective association between baseline ADL disability and hip fractures, expressed as odds ratios (OR) with 95% confidence intervals (CI). To mitigate the influence of confounding factors, we conducted multivariable adjusted models. Specifically, Model 1 is a simple regression model without adjustments for confounding factors. Model 2 adjusted for sex, age, marital status, residence, and education level; Model 3 further adjusted for drinking status, smoking status, chronic disease history (hypertension and diabetes), sleep duration, and BMI, while Model 4 additionally adjusted for a history of falls based on Model 3. Furthermore, we performed subgroup analyses to assess potential interactions among various variables, specifically examining interactions based on factors such as sex, age, marital status, residence, BMI, sleep duration, smoking status, drinking status, depressive symptoms, and chronic disease history (hypertension and diabetes).

The mediation model was constructed using the R package “mediation”, employing a modeling approach that utilizes Bootstrap with 1,000 simulations (34). This analysis assessed both the indirect and direct effects of ADL on the risk of hip fractures, as well as the mediation proportions attributed to the depression variable. Statistical analyses were conducted using R version 4.4.2, with a *p*-value threshold of less than 0.05 indicating statistical significance.

## 3 Result

### 3.1 Characteristics of the study population

This study included a total of 12,642 participants, with males representing 47.18% and females 52.82%. Participants were categorized into the normal function group and the disability group based on the IADL and BADL scales. The IADL disability group demonstrated significant differences compared to the normal group, including a higher median age, shorter sleep duration, lower BMI, a higher proportion of females, a lower proportion of married individuals, and a greater proportion of rural residents. Similarly, the BADL disability group exhibited notable differences from the normal group, characterized by older age, shorter sleep duration, a higher proportion of females and rural residents, while no intergroup difference in BMI was observed. Both groups of individuals with disability also displayed significant characteristics regarding educational level, risk behaviors, prevalence of chronic diseases, and incidence of falls: compared to the normal group, they exhibited lower educational levels, lower risk behaviors (with higher proportions of never smoking and never drinking), and a higher prevalence of chronic diseases (such as hypertension and diabetes), as well as a higher incidence of falls, with all comparisons yielding *p*-values less than 0.001 (except for BADL BMI). More detailed demographic characteristics are provided in Table 1.

**Table 1:** Baseline characteristics of participants categorized by ADL (2011).

Variables	Total	IDAL		*p*	BDAL		*p*
	Total (*n* = 12,642)	No (*n* = 10,076)	Yes (*n* = 2,566)		No (*n* = 10,667)	Yes (*n* = 1975)	
Age, median (IQR), years	58 (51, 65)	57 (51, 64)	61 (55.25, 69)	<0.001	57 (51, 64)	62 (56, 70)	<0.001
Sleep duration, median (IQR), hours	6 (5, 8)	7 (5, 8)	6 (4, 8)	<0.001	7 (5, 8)	6 (4, 7)	<0.001
Body mass index, median (IQR), kg/m^2^	23.11 (20.85, 25.75)	23.18 (20.94, 25.79)	22.8 (20.45, 25.58)	<0.001	23.11 (20.89, 25.67)	23.09 (20.64, 26.09)	0.987
Sex, *n* (%)				<0.001			<0.001
Female	6,677 (52.82)	5,075 (50.37)	1,602 (62.43)		5,491 (51.48)	1,186 (60.05)	
Male	5,965 (47.18)	5,001 (49.63)	964 (37.57)		5,176 (48.52)	789 (39.95)	
Marital status, *n* (%)				<0.001			<0.001
Others	1,509 (11.94)	1,049 (10.41)	460 (17.93)		1,161 (10.88)	348 (17.62)	
Married	11,133 (88.06)	9,027 (89.59)	2,106 (82.07)		9,506 (89.12)	1,627 (82.38)	
Educational level, *n* (%)				<0.001			<0.001
Illiteracy	5,858 (46.38)	4,164 (41.37)	1,694 (66.07)		4,591 (43.08)	1,267 (64.18)	
Primary school	2,727 (21.59)	2,259 (22.44)	468 (18.25)		2,358 (22.13)	369 (18.69)	
Middle school	2,613 (20.69)	2,316 (23.01)	297 (11.58)		2,369 (22.23)	244 (12.36)	
High school or above	1,432 (11.34)	1,327 (13.18)	105 (4.1)		1,338 (12.56)	94 (4.76)	
Residence, *n* (%)				<0.001			<0.001
Urban	4,643 (36.73)	3,931 (39.01)	712 (27.75)		4,102 (38.46)	541 (27.39)	
Rural	7,999 (63.27)	6,145 (60.99)	1854 (72.25)		6,565 (61.54)	1,434 (72.61)	
Smoking, *n* (%)				<0.001			<0.001
Ex-smoking	1,014 (8.02)	733 (7.28)	281 (10.96)		778 (7.3)	236 (11.96)	
Current smoking	4,153 (32.86)	3,484 (34.59)	669 (26.08)		3,640 (34.14)	513 (25.99)	
Never smoking	7,470 (59.11)	5,855 (58.13)	1,615 (62.96)		6,245 (58.57)	1,225 (62.06)	
Drinking, *n* (%)				<0.001			<0.001
Ex-drinking	1,049 (8.3)	804 (7.98)	245 (9.55)		847 (7.94)	202 (10.23)	
Current drinking	3,899 (30.84)	3,263 (32.39)	636 (24.79)		3,400 (31.88)	499 (25.27)	
Never drinking	7,693 (60.86)	6,008 (59.63)	1,685 (65.67)		6,419 (60.18)	1,274 (64.51)	
Hypertension, *n* (%)				<0.001			<0.001
No	6,923 (54.94)	5,672 (56.43)	1,251 (49.04)		5,971 (56.12)	952 (48.5)	
Yes	5,679 (45.06)	4,379 (43.57)	1,300 (50.96)		4,668 (43.88)	1,011 (51.5)	
Diabetes, *n* (%)				0.001			<0.001
No	11,007 (87.82)	8,824 (88.29)	2,183 (85.94)		9,353 (88.39)	1,654 (84.73)	
Yes	1,527 (12.18)	1,170 (11.71)	357 (14.06)		1,229 (11.61)	298 (15.27)	
Fall, *n* (%)				<0.001			<0.001
No	10,667 (84.41)	8,733 (86.71)	1934 (75.37)		9,239 (86.65)	1,428 (72.3)	
Yes	1970 (15.59)	1,338 (13.29)	632 (24.63)		1,423 (13.35)	547 (27.7)	

### 3.2 The longitudinal association between DAL and new-onset hip fractures

During the four-year follow-up period, a total of 256 respondents experienced hip fractures. In the adjusted model, the association between IADL and incident hip fractures remained significant, with an odds ratio (OR) of 1.42 (95% CI: 1.07–1.89), *p* = 0.017. Similarly, in the adjusted model, the association between BADL and the study outcome was also significant, with an OR of 1.96 (95% CI: 1.47–2.61), *p* < 0.001. These findings indicate that after adjusting for confounding factors such as sex, age, marital status, educational level, residence, chronic diseases, sleep duration, BMI, history of falls, drinking status, and smoking status, both IADL and BADL significantly predicted the study outcomes (Table 2).

**Table 2:** Presents the OR values and 95% confidence intervals for new-onset hip fractures based on ADL disability.

Variables	Model 1		Model 2		Model 3		Model 4	
	OR (95%CI)	*P*	OR (95%CI)	*P*	OR (95%CI)	*P*	OR (95%CI)	*P*
BADL								
No	1.00 (Reference)		1.00 (Reference)		1.00 (Reference)		1.00 (Reference)	
Yes	2.42 (1.85 ~ 3.18)	<0.001	2.04 (1.54 ~ 2.71)	<0.001	2.05 (1.54 ~ 2.73)	<0.001	1.96 (1.47 ~ 2.61)	<0.001
IADL								
No	1.00 (Reference)		1.00 (Reference)		1.00 (Reference)		1.00 (Reference)	
Yes	1.78 (1.36 ~ 2.32)	<0.001	1.47 (1.11 ~ 1.95)	0.008	1.48 (1.11 ~ 1.96)	0.007	1.42 (1.07 ~ 1.89)	0.017

The data are presented in the form of OR (95% CI). OR, odds ratio; CI, confidence interval; IADL, Instrumental Activities of Daily Living; BADL, Basic Activities of Daily Living.

Model 1 was unadjusted.

Model 2 was adjusted for sex, age, marital status, educational level, and residence.

Model 3 was further adjusted for hypertension, diabetes, sleep duration, BMI, drinking status, and smoking status based on Model 2.

Model 4 additionally adjusted for fall.

### 3.3 Subgroup analysis

**Figure 2:**
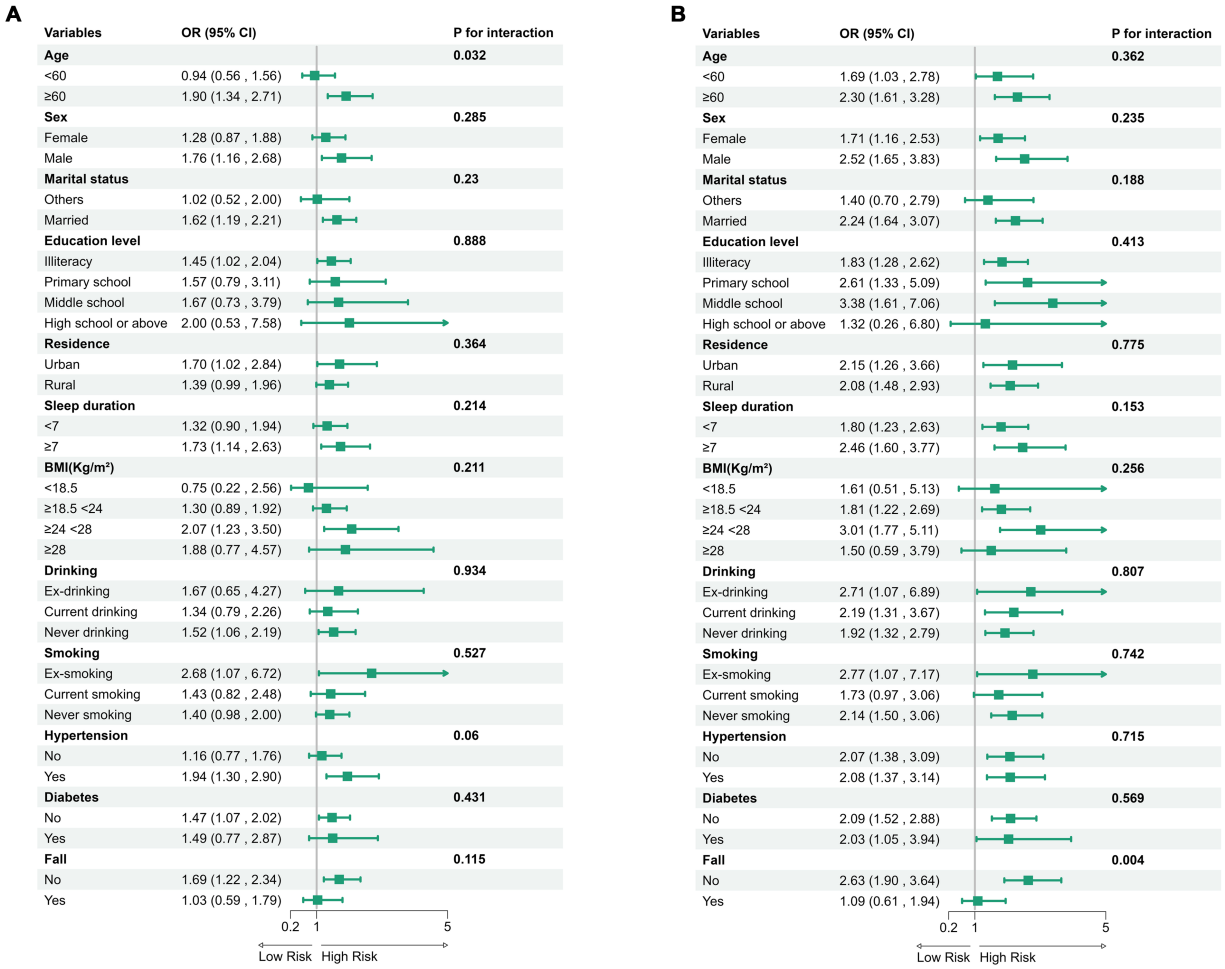
Subgroup analysis of IADL **(A)** and BADL **(B)** with the odds ratio (95% CI) of hip fractures.

To investigate the robustness of ADL and hip fractures, we divided participants into various subgroups. Further interaction tests indicated that age served as the primary moderating factor in the relationship between IADL and hip fractures (interaction *p* = 0.032), while a history of falls was the main moderator in the relationship between BADL and hip fractures (interaction *p* = 0.004) (Figure 2).

In the multivariate model, confounding factors such as age, sex, residence, educational level, BMI, sleep duration, drinking status, smoking status, history of chronic diseases (diabetes, hypertension), and history of falls were considered.

### 3.4 Mediation analysis

To further evaluate the mediating role of depressive states in the relationship between ADL disability and the risk of hip fractures, this study employed the Center for CESD-10 as a mediating variable, while controlling for factors such as age, sex, residence, education level, BMI, sleep duration, drinking status, smoking status, and chronic disease history, including diabetes and hypertension. The results indicate that depressive symptoms play a distinct mediating role between ADL disability and the risk of hip fractures. Statistical analyses reveal that the *p*-value for the average direct effect (ADE) of IADL on hip fracture risk is 0.21, which does not achieve statistical significance. This finding suggests that the direct impact of IADL on hip fracture risk is not significant, and its effect is primarily mediated by depressive symptoms. In other words, depressive symptoms serve as a complete mediator between IADL and hip fractures risk, with a mediating effect proportion as high as 43.8%. Conversely, the ADE *p*-value of BADL on hip fracture risk is less than 0.001, indicating high statistical significance. These results suggest that BADL not only has a significant direct impact on hip fractures risk but also that depressive symptoms exert a partial mediating effect between the two, with a mediating effect proportion of 23.4% (Table 3).

**Table 3:** Depressive symptoms mediating the association between IADL and BADL with hip fractures.

Depressive symptoms-ADL-Hip fractures	Effect	Estimate	95% CI lower	95% CI upper	*P*-value
IDAL	ACME (average)	0.0032	0.0016	0.005	<0.001 ***
	ADE (average)	0.0041	−0.0022	0.01	0.21
	Prop. Mediated (average)	0.4377	0.1663	2.39	0.016 *
BDAL	ACME (average)	0.0037	0.0017	0.01	<0.001 ***
	ADE (average)	0.0122	0.0046	0.02	<0.001 ***
	Prop. Mediated (average)	0.2342	0.1031	0.47	<0.001 ***

CI, confidence interval; IADL, Instrumental Activities of Daily Living; BADL, Basic Activities of Daily Living; ADE, average direct effect; ACME, average causal mediation effect.

## 4 Discussion

This study investigates the relationship between disability in ADL and hip fractures among middle-aged and older adult individuals in China, revealing a significant correlation between the two. To our knowledge, this is the first study to examine the mediating role of depressive symptoms in the relationship between ADL disability and hip fractures. Over a four-year follow-up period, we observed a total of 256 new cases of hip fractures. After controlling for factors such as sex, age, and marital status, the study found that disability in IADL was significantly associated with an increased risk of hip fractures (OR = 1.42, 95% CI: 1.07–1.89), while the impact of disability in BADL was even more pronounced (OR = 1.96, 95% CI: 1.47–2.61). Furthermore, mediation analysis indicated that depressive symptoms played a mediating role in this relationship: 43.8% of the effect of IADL disability on the risk of hip fractures was mediated through depressive symptoms, while for BADL disability, 23.4% of the effect was partially mediated by depressive symptoms. Our mediation analysis results suggest that addressing depressive symptoms may help mitigate the negative impact of functional disability on the risk of hip fractures.

This study, utilizing data from the CHARLS, confirms a positive correlation between ADL, encompassing both BADL and IADL, and the risk of hip fractures among the older adult. This finding aligns with multiple studies (10, 35, 36). Research conducted in the United States indicates that difficulties in performing daily living activities, including challenges with indoor walking, dressing, and bathing, are associated with an increased risk of hip fractures (36). Furthermore, an analysis from Korea reveals that the incidence of hip fractures among older adult individuals with disability is 1.6 times higher than that of their non-disabled counterparts (35). A meta-analysis conducted in Europe demonstrates that prior to experiencing hip fractures, older adult individuals exhibit significantly lower scores in daily living abilities compared to their non-fractured peers, suggesting that functional decline may precede fractures (10). Additionally, the HRS confirms that many patients who sustain hip fractures had already experienced severe disability prior to the fractures and relied on others for assistance with daily activities (11, 12).

Our research indicates that limitations in ADL disability are a significant factor contributing to an increased risk of hip fractures in the older adult. Firstly, ADL disability directly leads to reduced levels of physical activity, which accelerates the loss of skeletal muscle mass and strength, thereby inducing or exacerbating sarcopenia. Studies have shown that ADL disability is closely associated with a decrease in skeletal muscle mass, particularly in the older adult population, where diminished activity due to ADL disability directly affects the mechanical load on muscles, consequently accelerating muscle atrophy and strength decline (37). This loss of muscle mass not only diminishes mobility but also exacerbates skeletal fragility, especially in the hip region, thereby increasing the risk of fractures (38). Sarcopenia is a syndrome characterized by a decline in muscle mass and function associated with aging, and its occurrence is closely related to limitations in physical function (39). Research indicates that individuals with ADL restrictions often exhibit lower grip strength and lower limb muscle strength, which are highly consistent with the diagnostic criteria for sarcopenia (40). Longitudinal data from CHARLS confirm that the risk of hip fractures in sarcopenic patients is twice that of non-sarcopenic individuals, and this association is independent of traditional risk factors (41). It is noteworthy that there exists a significant interaction between activities of ADL disability and the decline in grip strength. ADL disability are associated with reduced mobility and grip strength, which are critical indicators of muscle degradation (42). Grip strength, recognized as a core diagnostic criterion for sarcopenia, correlates with diminished dynamic balance ability and an elevated risk of falls (43, 44). Concurrently, research suggests that a decline in grip strength itself can lead to an increased risk of hip fractures, serving as a vital tool for predicting both fractures and fall risk (32, 45). Furthermore, individuals with ADL disability, particularly those with BDAL disability, encounter challenges related to insufficient intensity in their daily activities. This insufficiency can reduce bone density and contribute to the development of osteoporosis, thus heightening the risk of hip fractures. Studies have demonstrated a significant negative correlation between levels of physical activity and bone mineral density (BMD), as well as fracture risk: higher physical activity levels are associated with a decreased risk of hip fractures, while sedentary behavior is closely linked to an increased risk (46–48). Additionally, inadequate intensity of daily activities among the older adult can directly diminish the mechanical load on bones, adversely affecting bone metabolism and exacerbating bone density decline, which further increases the risk of hip fractures (49, 50). Lastly, the cessation of physical activity among the older adult, prompted by fear of falling or physical limitations, intensifies bone loss and raises the risk of hip fractures (51).

This study reveals, for the first time through mediation analysis, that disability in ADL may indirectly increase the risk of hip fractures by inducing or exacerbating depressive symptoms. This finding is supported by research derived from the CHARLS, which indicates that impaired ADL directly elevates the risk of hip fractures. Additionally, baseline depressive symptoms are associated with an increased risk of hip fractures, and this association is fully mediated by cognitive decline and ADL disability (52). Notably, this study does not elaborate on the mediating mechanism of ADL disability in the occurrence of hip fractures. Based on this, we propose that depressive symptoms serve as a more appropriate mediating factor linking ADL disability and hip fractures. Our research further confirms that depressive symptoms play a crucial mediating role in the pathway through which ADL disability affect the risk of hip fractures.

The exacerbation of depressive symptoms has been confirmed to accelerate muscle atrophy and abnormalities in bone metabolism, both of which are crucial factors that elevate the risk of hip fractures (53). Sarcopenia, a common consequence of reduced physical activity, may be intensified by the physiological stress associated with depression, potentially leading to enhanced catabolic processes and decreased muscle protein synthesis (8). The relationship between depression and osteoporosis in postmenopausal women is significant, as they face an elevated risk of bone loss due to declining estrogen levels. Studies indicate that the bone mineral density of women with depression is significantly lower than that of their non-depressed counterparts, resulting in a higher incidence of osteoporosis (54, 55). The interaction between depressive symptoms and musculoskeletal health is particularly pronounced in the older adult, who are at an increased risk for both depressions and bone-related injuries (56). The bidirectional relationship between disability in ADL and depressive symptoms has been extensively studied. On one hand, individuals with ADL disability are more likely to exhibit depressive symptoms due to the psychological stress stemming from the loss of independence and reliance on others for daily activities (57). Conversely, depressive symptoms may lead to a decline in physical function, further exacerbating ADL disability (58). This mutual influence creates a feedback loop that can significantly impair an individual’s quality of life and increase the risk of adverse health outcomes, such as hip fractures (59). Specifically, ADL disability can lead to reduced social participation and feelings of helplessness, which may exacerbate depressive symptoms. The worsening of these symptoms can further accelerate muscle atrophy and deterioration of bone metabolism, thereby increasing the risk of hip fractures (60–62). Longitudinal studies based on the CHARLS have confirmed a significant bidirectional association between ADL disability and depressive symptoms among the middle-aged and older adult population (16, 63). Furthermore, previous research has demonstrated that depression is associated with a decrease in bone mineral density, which increases the risk of osteoporotic fractures. Notably, the risk of hip fractures in depressed patients is significantly higher than that in non-depressed individuals (52, 64).

Depression, along with its associated chronic inflammatory response and dysfunction of the hypothalamic–pituitary–adrenal (HPA) axis, has garnered significant attention due to its substantial negative impact on bone health, particularly concerning the risk of hip fractures (65). The chronic activation of the HPA axis, commonly observed in patients with depression, results in sustained elevations in cortisol levels, which disrupt bone metabolism and promote bone loss (66, 67). Specifically, cortisol impairs bone formation and enhances bone resorption by inhibiting osteoblast activity, promoting osteoclastogenesis, and reducing calcium absorption (68, 69). Concurrently, inflammatory cytokines involved in the pathophysiology of depression, such as IL-6 and TNF-*α*, directly affect bone metabolism by stimulating osteoclast activity and inhibiting osteoblast function, thereby disrupting bone homeostasis (70, 71). Moreover, a vicious cycle exists between HPA axis dysfunction and inflammatory pathways: elevated pro-inflammatory cytokines (such as IL-6 and TNF-α) exacerbate HPA axis dysregulation and its detrimental effects on bone, while HPA axis activation further promotes the inflammatory response, collectively aggravating the imbalance of bone homeostasis (72, 73). Additionally, in patients with depression, HPA axis dysfunction can lead to increased glucocorticoids, which inhibit osteoblast function and promote osteoclast activity, thereby exacerbating bone loss (74, 75). This pathological process, driven by depression and related neuroendocrine-immune dysregulation, significantly increases the risk of hip fractures in middle-aged and older adult individuals.

Based on this, it is essential to develop differentiated intervention strategies tailored to the various types of ADL disability. For individuals with IADL disability, the focus should be on psychological adjustment and the management of depressive symptoms to enhance their daily living activities. In contrast, for patients experiencing BADL disability, intervention measures should prioritize physical exercise, as regular training can significantly enhance bone density (76). Beyond improving BMD and bone strength, physical exercise also contributes to increased muscle mass and strength, thereby reducing the risk of falls and hip fractures (77). In clinical practice, it is crucial to prioritize the assessment of ADL functionality and the screening for depressive states to develop individualized intervention plans, ultimately aiming to reduce the risk of hip fractures among the middle-aged and older adult population.

This study is based on the CHARLS study, a nationally representative longitudinal cohort study, which offers several advantages. First, the sample size is sufficiently large to ensure robust statistical power, and CHARLS provides national sample representation. Second, throughout the study, we adjusted for confounding variables to enhance the reliability of the results. By introducing depressive symptoms as a mediator, this study attempts for the first time to establish the association between depressive symptoms, ADL disability, and the risk of hip fractures in the Chinese population. However, there are limitations that should be acknowledged. First, the assessment of hip fractures relies entirely on self-reports rather than radiological or clinical data, which may lead to recall bias and result in the exclusion of some subjects with hip fractures, potentially underestimating the actual incidence. Nonetheless, the interviewer provided detailed descriptions of the hip bone location to enhance the accuracy of respondents’ answers. Second, the CES-D10 questionnaire was administered through self-reports from the sample population, which may introduce the risk of recall bias and inaccurate responses. Although the CES-D10 is a widely used tool for assessing clinically significant depressive symptoms, it serves only as a screening tool and cannot be used for diagnosing depression. Fourth, due to the absence of certain key potential fracture risk factors in the CAHRLS dataset, we are unable to fully adjust for all risk factors associated with hip fractures, including pre-existing osteoporosis/osteopenia, sarcopenia, bone mineral density, and medication treatment status. Finally, this study utilized a representative sample from the national population of China; therefore, caution should be exercised when generalizing the findings to other populations.

## 5 Conclusion

This study of CHARLS data (2011–2015) shows that ADL, especially BADL disability, predicts hip fractures in older Chinese adults, with depressive symptoms mediating 43.8% of IADL and 23.4% of BADL associations with fractures. Integrating depression management into ADL interventions could improve fracture prevention. The findings highlight the importance of addressing ADL disability and depression in risk strategies, suggesting future research on their link and interventions to enhance ADL function and mental health.

## Data Availability

Publicly available datasets were analyzed in this study. This data can be found here: publicly available datasets were analyzed in this study. This data can be found at: http://charls.pku.edu.cn/pages/data/111/zh-cn.html.

## References

[1] Hai Y Zhidong C Wenyan W. Human umbilical cord mesenchymal stromal cells promotes the proliferation and osteogenic differentiation of autologous bone marrow stem cells by secreting exosomes. Bioengineered. (2022) 13:9901–15. doi: 10.1080/21655979.2022.2062183 35412945 PMC9162006

[2] Lin X Guo H Lian Y Kou J Wang G Chen Y . Osteoporosis and related health status among the elderly urban residents in elderly-care inns in Beijing, a Multicenter DXA survey. Front Endocrinol (Lausanne). (2022) 13:875678. doi: 10.3389/fendo.2022.875678, PMID: 35957840 PMC9359074

[3] Vandenput L Kindblom JM Bygdell M Nethander M Ohlsson C. Pubertal timing and adult fracture risk in men: a population-based cohort study. PLoS Med. (2019) 16:e1002986. doi: 10.1371/journal.pmed.1002986, PMID: 31790400 PMC6886748

[4] Yang W Li G Liu J. The incidence, prevalence, and health burden of hip fractures in China: data from the global burden of disease study 2019. Prev Med Rep. (2024) 38:102622. doi: 10.1016/j.pmedr.2024.102622, PMID: 38375171 PMC10874847

[5] Feng G Tingrun C Yufeng G Gang L Zhelun T Yimin C . Epidemiological trends and mid-term to long-term outcomes of acetabular fractures in the elderly in China. Int Orthop. (2024) 48:563–72. doi: 10.1007/s00264-023-06032-0, PMID: 38019297 PMC10799810

[6] Peng J Ye P Zhang J Zhang X Peng K He J . Characteristics of falls among older hip fracture patients from six Chinese hospitals: a post-hoc descriptive analysis. BMC Geriatr. (2023) 23:284. doi: 10.1186/s12877-023-03971-6, PMID: 37170210 PMC10176772

[7] Sarah H Nuno M Terry A Andrew K Carmen R-M Carol J . The association between 25-hydroxyvitamin D concentration and disability trajectories in very old adults: the Newcastle 85+ study. Nutrients. (2020) 12:2742. doi: 10.3390/nu12092742, PMID: 32916847 PMC7551468

[8] Hu Y Zhou F Kaminga AC Yan S Hu Z. Associations of depressive symptoms and chronic diseases with activities of daily living among middle-aged and older population in China: A population-based cohort study. Front Psych. (2022) 13:848255. doi: 10.3389/fpsyt.2022.848255, PMID: 36003971 PMC9393545

[9] John PA Chi-Tsun C Aloysius Chia W-Y Tessa Lui Shi M David BM. Trends in functional disability and cognitive impairment among the older adult in China up to 2060: estimates from a dynamic multi-state population model. BMC Geriatr. (2021) 21:380. doi: 10.1186/s12877-021-02309-4, PMID: 34157986 PMC8218480

[10] AK S IS C W JB CS R ML W KE C. Increase in disability prevalence before hip fracture. J Am Geriatr Soc. (2015) 63:2029–35. doi: 10.1111/jgs.13658, PMID: 26480970 PMC4699653

[11] Ravensbergen WM Blom JW Kingston A Robinson L Kerse N Teh RO . Declining daily functioning as a prelude to a hip fracture in older persons-an individual patient data meta-analysis. Age Ageing. (2022) 51:afab253. doi: 10.1093/ageing/afab253, PMID: 35077559 PMC8789300

[12] Tang VL Sudore R Cenzer IS Boscardin WJ Smith A Ritchie C . Rates of recovery to pre-fracture function in older persons with hip fracture: an observational study. J Gen Intern Med. (2017) 32:153–8. doi: 10.1007/s11606-016-3848-2, PMID: 27605004 PMC5264672

[13] Chang-Quan H Xue-Mei Z Bi-Rong D Zhen-Chan L Ji-Rong Y Qing-Xiu L. Health status and risk for depression among the elderly: a meta-analysis of published literature. Age Ageing. (2010) 39:23–30. doi: 10.1093/ageing/afp187, PMID: 19903775

[14] Shao M Chen J Ma C. Research on the relationship between Chinese elderly health status, social security, and depression. Int J Environ Res Public Health. (2022) 19:7496. doi: 10.3390/ijerph19127496, PMID: 35742744 PMC9223444

[15] Jiang J Tang Z Futatsuka M. The impact of ADL disability on depression symptoms in a community of Beijing elderly, China. Environ health. Prev Med. (2002) 7:199–204. doi: 10.1007/bf02898005, PMID: 21432278 PMC2723587

[16] Wang S Yu M Huang W Wang T Liu K Xiang B. Longitudinal association between ADL disability and depression in middle-aged and elderly: national cohort study. J Nutr Health Aging. (2025) 29:100450. doi: 10.1016/j.jnha.2024.100450, PMID: 39674106 PMC12180056

[17] Feng Z Li Q Zhou L Chen Z Yin W. The relationship between depressive symptoms and activity of daily living disability among the elderly: results from the China health and retirement longitudinal study (CHARLS). Public Health. (2021) 198:75–81. doi: 10.1016/j.puhe.2021.06.023, PMID: 34365109

[18] Kim SY Lee JK Oh DJ Kong IG Choi HG. Depression and incident hip fracture: a longitudinal follow-up study using a national sample cohort. Medicine (Baltimore). (2019) 98:e16268. doi: 10.1097/md.0000000000016268, PMID: 31261597 PMC6617478

[19] Lian Z Zhu C Yuan H Wang J. Association between changes in depressive symptoms and hip fracture among middle-aged and older Chinese individuals: a prospective cohort study. BMC Geriatr. (2022) 22:844. doi: 10.1186/s12877-022-03484-8, PMID: 36348273 PMC9644634

[20] Hussain MA Qaisar R Karim A Ahmad F Franzese F Awad A . Predictors of hip fracture in 15 European countries: a longitudinal study of 48,533 geriatric adults using SHARE dataset. Arch Osteoporos. (2024) 19:60. doi: 10.1007/s11657-024-01420-4, PMID: 39023661

[21] Pan CC Hu LY Lu T Tu MS Shen CC Chen ZJ. Risk of hip fractures in patients with depressive disorders: a nationwide, population-based, retrospective, cohort study. PLoS One. (2018) 13:e0194961. doi: 10.1371/journal.pone.0194961, PMID: 29641581 PMC5894998

[22] Gao K Ma WZ Huck S Li BL Zhang L Zhu J . Association between sarcopenia and depressive symptoms in Chinese older adults: evidence from the China health and retirement longitudinal study. Front Med. (2021) 8:755705. doi: 10.3389/fmed.2021.755705, PMID: 34869454 PMC8635632

[23] Chen Z Ho M Chau PH. Handgrip strength asymmetry is associated with the risk of neurodegenerative disorders among Chinese older adults. J Cachexia Sarcopenia Muscle. (2022) 13:1013–23. doi: 10.1002/jcsm.12933, PMID: 35178892 PMC8977973

[24] Du G Fan Z Fan K Liu H Zhang J Li D . Risk-stratified lifetime risk and incidence of hip fracture and falls in middle-aged and elderly Chinese population: the China health and retirement longitudinal study. J Orthop Translat. (2025) 50:174–84. doi: 10.1016/j.jot.2024.10.013, PMID: 39895863 PMC11782876

[25] Mei F Li JJ Lin J Xing D Dong S. Multidimensional characteristics of musculoskeletal pain and risk of hip fractures among elderly adults: the first longitudinal evidence from CHARLS. BMC Musculoskelet Disord. (2024) 25:4. doi: 10.1186/s12891-023-07132-z, PMID: 38166800 PMC10759596

[26] Qiao Y Liu S Li G Lu Y Wu Y Shen Y . Longitudinal follow-up studies on the bidirectional association between ADL/IADL disability and multimorbidity: results from two national sample cohorts of middle-aged and elderly adults. Gerontology. (2021) 67:563–71. doi: 10.1159/000513930, PMID: 34182559

[27] Zhang J Huang H Lin Z Dong J Zhang X Gao J . Associations between cardiovascular-kidney-metabolic syndrome and disability in activities of daily living: a nationwide longitudinal study among the middle-aged and older adults in China. Front Public Health. (2024) 12:1480576. doi: 10.3389/fpubh.2024.1480576 39882115 PMC11774716

[28] Wang KC Lo YC Liao CC Jou YY Huang HB. Associations between symptoms of depression and air pollutant exposure among older adults: results from the Taiwan longitudinal study on aging (TLSA). Front Public Health. (2021) 9:779192. doi: 10.3389/fpubh.2021.779192 35096739 PMC8790292

[29] Wijayabahu AT Mickle AM Mai V Garvan C Glover TL Cook RL . Associations between vitamin D, omega 6:omega 3 ratio, and biomarkers of aging in individuals living with and without chronic pain. Nutrients. (2022) 14:266. doi: 10.3390/nu14020266, PMID: 35057447 PMC8779718

[30] Liu Y Cui J Cao L Stubbendorff A Zhang S. Association of depression with incident sarcopenia and modified effect from healthy lifestyle: the first longitudinal evidence from the CHARLS. J Affect Disord. (2024) 344:373–9. doi: 10.1016/j.jad.2023.10.012, PMID: 37805156

[31] Li C Zhao X Zhang L Ma C Zhang W Ding H. Anemia as a mediator: bridging the frailty index and hip fractures in older Chinese populations. Front Public Health. (2025) 13:1558074. doi: 10.3389/fpubh.2025.1558074, PMID: 40337739 PMC12055539

[32] Zhou S Si H Wu L Liu Y Peng L Li M . Association between handgrip strength weakness and asymmetry with incident hip fracture among older Chinese adults. Arch Gerontol Geriatr. (2024) 122:105385. doi: 10.1016/j.archger.2024.105385, PMID: 38417298

[33] Song J Wu X Zhang Y Song P Zhao Y. Association between changes in depressive symptoms and falls: the China health and retirement longitudinal study (CHARLS). J Affect Disord. (2023) 341:393–400. doi: 10.1016/j.jad.2023.09.004, PMID: 37683944

[34] Qian T Sheng X Shen P Fang Y Deng Y Zou G. Mets-IR as a predictor of cardiovascular events in the middle-aged and elderly population and mediator role of blood lipids. Front Endocrinol (Lausanne). (2023) 14:1224967. doi: 10.3389/fendo.2023.1224967, PMID: 37534205 PMC10393118

[35] Kim J Jang SN Lim JY. Pre-existing disability and its risk of fragility hip fracture in older adults. Int J Environ Res Public Health. (2019) 16:1237. doi: 10.3390/ijerph16071237, PMID: 30959977 PMC6480526

[36] Wilson RT Chase GA Chrischilles EA Wallace RB. Hip fracture risk among community-dwelling elderly people in the United States: a prospective study of physical, cognitive, and socioeconomic indicators. Am J Public Health. (2006) 96:1210–8. doi: 10.2105/ajph.2005.077479, PMID: 16735617 PMC1483878

[37] Han S Shin S Kim O Hong N. Characteristics associated with bone loss after spinal cord injury: implications for hip region vulnerability. Endocrinol Metab (Seoul). (2023) 38:578–87. doi: 10.3803/EnM.2023.1795, PMID: 37816499 PMC10613772

[38] Islamoglu I Çebi M Tosun FC. The bone mineral density and isokinetic knee strength in amputee soccer players. Rev Assoc Med Bras (1992). (2023) 69:e20230100. doi: 10.1590/1806-9282.20230100, PMID: 37585984 PMC10427187

[39] Huang Y Zhu X Chen K Lang H Zhang Y Hou P . Resveratrol prevents sarcopenic obesity by reversing mitochondrial dysfunction and oxidative stress via the PKA/LKB1/AMPK pathway. Aging (Albany NY). (2019) 11:2217–40. doi: 10.18632/aging.101910, PMID: 30988232 PMC6519996

[40] Takamoto K Morizaki Y Fukuda A Ohe T. Hand grip strength differences in geriatric subjects with and without hand diseases. Prog Rehabil Med. (2023) 8:20230030. doi: 10.2490/prm.20230030, PMID: 37736258 PMC10509485

[41] Luo C Liu R Shen X Zhang G Liu B. Possible sarcopenia and risk of hip fracture in older adults in China. Arch Gerontol Geriatr. (2024) 117:105248. doi: 10.1016/j.archger.2023.105248, PMID: 37897854

[42] Dai S Wang S Jiang S Wang D Dai C. Bidirectional association between handgrip strength and ADLs disability: a prospective cohort study. Front Public Health. (2023) 11:1200821. doi: 10.3389/fpubh.2023.1200821, PMID: 37663846 PMC10470652

[43] Kirk B Zanker J Bani Hassan E Bird S Brennan-Olsen S Duque G . Sarcopenia definitions and outcomes consortium (SDOC) criteria are strongly associated with malnutrition, depression, falls, and fractures in high-risk older persons. J Am Med Dir Assoc. (2021) 22:741–5. doi: 10.1016/j.jamda.2020.06.050, PMID: 32771358

[44] Lim SK Kong S. Prevalence, physical characteristics, and fall risk in older adults with and without possible sarcopenia. Aging Clin Exp Res. (2022) 34:1365–71. doi: 10.1007/s40520-022-02078-z, PMID: 35133613

[45] Tianting G Fei Z Lijiao X Zhihua H Xiaoan Z Junming W . Association of handgrip strength with hip fracture and falls in community-dwelling middle-aged and older adults: a 4-year longitudinal study. Orthop Surg. (2024) 16:1051–63. doi: 10.1111/os.14029, PMID: 38485456 PMC11062856

[46] Lagerros YT Hantikainen E Michaëlsson K Ye W Adami HO Bellocco R. Physical activity and the risk of hip fracture in the elderly: a prospective cohort study. Eur J Epidemiol. (2017) 32:983–91. doi: 10.1007/s10654-017-0312-5, PMID: 28940092 PMC5684287

[47] LaMonte MJ Wactawski-Wende J Larson JC Mai X Robbins JA LeBoff MS . Association of Physical Activity and Fracture Risk among Postmenopausal Women. JAMA Netw Open. (2019) 2:e1914084. doi: 10.1001/jamanetworkopen.2019.14084, PMID: 31651972 PMC6822158

[48] Rong K Liu XY Wu XH Li XL Xia QQ Chen J . Increasing level of leisure physical activity could reduce the risk of hip fracture in older women: A dose-response meta-analysis of prospective cohort studies. Medicine (Baltimore). (2016) 95:e2984. doi: 10.1097/md.0000000000002984 26986111 PMC4839892

[49] Hsu CY Huang CY Hsieh CH Chien PC Chen CC Hou SY . Regular exercise and weight-control behavior are protective factors against osteoporosis for general population: a propensity score-matched analysis from Taiwan biobank participants. Nutrients. (2022) 14:641. doi: 10.3390/nu14030641, PMID: 35277000 PMC8838409

[50] Movaseghi F Sadeghi H. Effect of three-year multi-component exercise training on bone mineral density and content in a postmenopausal woman with osteoporosis: A case report. Iran J Public Health. (2015) 44:701–4. PMID: 26284213 PMC4537629

[51] Villette CC Phillips ATM. Influence of a change in activity regime on femoral bone architecture and failure behaviour. PLoS One. (2024) 19:e0297932. doi: 10.1371/journal.pone.0297932, PMID: 38683797 PMC11057758

[52] Wu S Shi H Cheng R Xiang Z Huang SS. Impairment in activities of daily living and cognitive decline mediate the association between depressive symptoms and incident hip fractures in Chinese older adults. Bone. (2022) 159:116374. doi: 10.1016/j.bone.2022.116374, PMID: 35227932

[53] Yu J Zhu H Zhang Y Wang D Guo H Liu X . The relationship between dysphagia and frailty among Chinese hospitalized older patients: a serial mediation model through self-perceived oral health and self-reported nutritional status. BMC Geriatr. (2024) 24:110. doi: 10.1186/s12877-024-04684-0, PMID: 38287262 PMC10826207

[54] Bener A Saleh NM Bhugra D. Depressive symptoms and bone mineral density in menopause and postmenopausal women: a still increasing and neglected problem. J Family Med Prim Care. (2016) 5:143–9. doi: 10.4103/2249-4863.184640, PMID: 27453860 PMC4943122

[55] Okereke OI Reynolds CF Mischoulon D Chang G Vyas CM Cook NR . Effect of long-term vitamin D3 supplementation vs placebo on risk of depression or clinically relevant depressive symptoms and on change in mood scores: a randomized clinical trial. JAMA. (2020) 324:471–80. doi: 10.1001/jama.2020.10224, PMID: 32749491 PMC7403921

[56] Hoffman GJ Hays RD Wallace SP Shapiro MF Ettner SL. Depressive symptomatology and fall risk among community-dwelling older adults. Soc Sci Med. (2017) 178:206–13. doi: 10.1016/j.socscimed.2017.02.020, PMID: 28279573 PMC5411980

[57] Marconcin P Peralta M Ferrari G Gaspar de Matos M Espanha M Murawska-Ciałowicz E . The Association of Grip Strength with depressive symptoms among middle-aged and older adults with different chronic diseases. Int J Environ Res Public Health. (2020) 17:6942. doi: 10.3390/ijerph17196942, PMID: 32977410 PMC7579263

[58] DeFalco A Dolezal L Holt R Murray S Pullin G. Imagining technologies for disability futures. Lancet. (2022) 399:1772–3. doi: 10.1016/s0140-6736(22)00781-4, PMID: 35526545

[59] Lane R. Dinesh Palipana: Clinician and leading disability rights advocate. Lancet. (2022) 400:803. doi: 10.1016/s0140-6736(22)01697-x, PMID: 36088941

[60] Fu X Su Y Zeng C Liu L Guo Y Wu Y. The mediation and interaction of depressive symptoms in activities of daily living and active aging in rural elderly: A cross-sectional survey. Front Public Health. (2022) 10:942311. doi: 10.3389/fpubh.2022.942311, PMID: 36187612 PMC9517948

[61] Elbelt U Ahnis A Riedl A Burkert S Schuetz T Ordemann J . Associations of physical activity with depressiveness and coping in subjects with high-grade obesity aiming at bariatric surgery: a cross-sectional study. Biopsychosoc Med. (2015) 9:16. doi: 10.1186/s13030-015-0042-4, PMID: 26110016 PMC4479107

[62] Zhang Y Yang M Li M. Causality between sarcopenia-related traits and major depressive disorder: a bi-directional, two-sample mendelian randomized study. Medicine (Baltimore). (2023) 102:e35071. doi: 10.1097/md.0000000000035071, PMID: 37800817 PMC10553098

[63] Zhu X Wang Y Luo Y Ding R Shi Z He P. Bidirectional, longitudinal associations between depressive symptoms and IADL/ADL disability in older adults in China: a national cohort study. BMC Geriatr. (2024) 24:659. doi: 10.1186/s12877-024-05248-y, PMID: 39107705 PMC11301930

[64] Ustevic C Rajovic N Stanisavljevic D Tiosavljevic D Pavlovic A Tasic R . From sarcopenia to depressive symptoms in elderly: a path analysis. Int J Environ Res Public Health. (2023) 20:972. doi: 10.3390/ijerph20020972, PMID: 36673727 PMC9859183

[65] He MC Xia SH Pan H Zhou TT Wang XL Li JM . Chaihu-Shugan-San ameliorated osteoporosis of mice with depressive behavior caused by chronic unpredictable mild stress via repressing neuroinflammation and HPA activity. Drug Des Devel Ther. (2024) 18:5997–6015. doi: 10.2147/dddt.S480077, PMID: 39687683 PMC11648556

[66] Bilal H McDonald SJ Stout JC Harding IH. Associations of inflammatory cytokines and cortisol with nonmotor features of Huntington's disease. Ann Clin Transl Neurol. (2024) 11:989–99. doi: 10.1002/acn3.52016, PMID: 38356101 PMC11021624

[67] Steardo L Jr Luciano M Sampogna G Carbone EA Caivano V Di Cerbo A . Clinical severity and calcium metabolism in patients with bipolar disorder. Brain Sci. (2020) 10:417. doi: 10.3390/brainsci10070417, PMID: 32630307 PMC7408522

[68] Kim LU Chou T D'Orsogna MR. Onset, timing, and exposure therapy of stress disorders: mechanistic insight from a mathematical model of oscillating neuroendocrine dynamics. Biol Direct. (2016) 11:13. doi: 10.1186/s13062-016-0117-6, PMID: 27013324 PMC4807591

[69] Schorr M Lawson EA Dichtel LE Klibanski A Miller KK. Cortisol measures across the weight spectrum. J Clin Endocrinol Metab. (2015) 100:3313–21. doi: 10.1210/jc.2015-2078, PMID: 26171799 PMC4570173

[70] Damani JJ De Souza MJ Strock NCA Koltun KJ Williams NI Weaver C . Associations between inflammatory mediators and bone outcomes in postmenopausal women: A cross-sectional analysis of baseline data from the prune study. J Inflamm Res. (2023) 16:639–63. doi: 10.2147/jir.S397837, PMID: 36814438 PMC9939790

[71] Huang R Chen Y Tu M Wang W. Monocyte to high-density lipoprotein and apolipoprotein A1 ratios are associated with bone homeostasis imbalance caused by chronic inflammation in postmenopausal women with type 2 diabetes mellitus. Front Pharmacol. (2022) 13:1062999. doi: 10.3389/fphar.2022.1062999, PMID: 36419622 PMC9676449

[72] Mo L Ma C Wang Z Li J He W Niu W . Integrated bioinformatic analysis of the shared molecular mechanisms between osteoporosis and atherosclerosis. Front Endocrinol. (2022) 13:950030. doi: 10.3389/fendo.2022.950030, PMID: 35937806 PMC9353191

[73] Haffner-Luntzer M Foertsch S Fischer V Prystaz K Tschaffon M Mödinger Y . Chronic psychosocial stress compromises the immune response and endochondral ossification during bone fracture healing via β-AR signaling. Proc Natl Acad Sci USA. (2019) 116:8615–22. doi: 10.1073/pnas.1819218116, PMID: 30948630 PMC6486758

[74] Batista SL de Araújo IM Carvalho AL Alencar M Nahas AK Elias J . Beyond the metabolic syndrome: visceral and marrow adipose tissues impair bone quantity and quality in Cushing's disease. PLoS One. (2019) 14:e0223432. doi: 10.1371/journal.pone.0223432, PMID: 31613908 PMC6793883

[75] Azuma K Furuzawa M Fujiwara S Yamada K Kubo KY. Effects of active mastication on chronic stress-induced bone loss in mice. Int J Med Sci. (2015) 12:952–7. doi: 10.7150/ijms.13298, PMID: 26664256 PMC4661293

[76] Kopiczko A Łopuszańska-Dawid M Gryko K. Bone mineral density in young adults: the influence of vitamin D status, biochemical indicators, physical activity and body composition. Arch Osteoporos. (2020) 15:45. doi: 10.1007/s11657-020-0684-0, PMID: 32166587 PMC7067719

[77] Lionikaite V Henning P Drevinge C Shah FA Palmquist A Wikström P . Vitamin A decreases the anabolic bone response to mechanical loading by suppressing bone formation. FASEB J. (2019) 33:5237–47. doi: 10.1096/fj.201802040R, PMID: 30668919 PMC6436664

