# Correction: The impact of ADL disability in middle-aged and older adults on the incidence of hip fractures and the mediating role of depression: a longitudinal evidence from CHARLS

**DOI:** 10.3389/fmed.2026.1929037

**Published:** 2026-07-27

**Authors:** Chenyang Li, Congcong Luo, Jiayi Zhu, Ruonan You, Qiang Yuan, Ning Zhang, Ying Zhang

**Affiliations:** 1 Department of College of Orthopedics and Traumatology, Fujian University of Traditional Chinese Medicine, Fuzhou, Fujian, China; 2 Luoyang Orthopedic-Traumatological Hospital of Henan Province (Henan Provincial Orthopedic Hospital), Luoyang, China; 3 Department of Graduate School of Henan University of Chinese Medicine, Henan University of Traditional Chinese Medicine, Zhengzhou, Henan, China; 4 Zhengzhou Health College, Zhengzhou, Henan, China

**Keywords:** hip fractures, Activities of Daily Living (ADL), depressive symptoms, mediating role, older adults

Affiliation 1, Department of College of Orthopedics and Traumatology, Fujian University of Traditional Chinese Medicine, Fuzhou, Fujian, China, was omitted from the paper for author Ying Zhang. This affiliation has now been added for author Ying Zhang.

In addition, the name of Affiliation 2 should be standardized as: Luoyang Orthopedic-Traumatological Hospital of Henan Province (Henan Provincial Orthopedic Hospital), Luoyang, China.

The corrected affiliation for the corresponding author should read: Ying Zhang^1,2^^*^.

The authors confirm that this is a non-commercial academic affiliation and that the Conflict of Interest statement remains unchanged.

The original version of this article has been updated.

